# Control of Pulse Pressure and Factors Affecting it among the Geriatric Population Suffering from Hypertension within the Community

**DOI:** 10.31083/RCM26156

**Published:** 2025-02-18

**Authors:** Qianfeng Yang, Lishuang Xu, Qingxia Gao, Zhiguang Gao

**Affiliations:** ^1^Department of Cardiology, Graduate School of Chengde Medical University, 067000 Chengde, Hebei, China; ^2^Department of Cardiology, Shengjing Hospital of China Medical University, 110004 Shenyang, Liaoning, China; ^3^Department of General Practice, Chaoyang Central Hospital, 122000 Chaoyang, Liaoning, China

**Keywords:** elderly, hypertension, pulse pressure control, community physician intervention

## Abstract

**Background:**

To explore the impact of employing the Knowledge, Attitude, and Practice (KAP) model within a unified community physician intervention aimed at managing pulse pressure among elderly individuals with hypertension in Shenyang, along with its associated influencing variables.

**Methods:**

2660 hypertensive patients were recruited in the community of Shenyang City in January 2020. After a 1-year KAP intervention by a unified community physician, KAP changes and pulse pressure levels were compared before and after the intervention. Meanwhile, the relevant influences affecting pulse pressure control were explored. Descriptive analysis and multifactorial logistic regression were used.

**Results:**

A significant decrease in pulse pressure by 10.71 mmHg (95% CI: 10.09, 11.33 mmHg) was noted among elderly individuals with hypertension in the community after undergoing a rigorous one-year intervention program (t = 33.79,* p* < 0.05). Pulse pressure control increased from 32.59% at baseline to 64.92% (χ^2^ = 556.43, *p* < 0.01). Compared to pre-intervention, knowledge about hypertension, awareness of prevention, medication and behavioural adherence improved significantly. A multifactorial logistic regression analysis revealed that the risk factors for pulse pressure control were female sex, a history of comorbid diabetes mellitus and poor adherence to medication due to forgetfulness.

**Conclusions:**

Unified community physician interventions can change the perceptions of elderly hypertensive patients, improve medication adherence, and improve poor lifestyle habits, thereby improving pulse pressure control in the geriatric population with hypertension residing in local communities.

## 1. Introduction

Hypertension is defined as an elevation in arterial pressure within the body’s circulatory system. This elevation may result in functional and structural damage to multiple organs, including the heart and brain. Ultimately, this damage may lead to organ failure [1]. This elevation is often accompanied by an increase in pulse pressure (PP), which is defined as the variance between systolic and diastolic blood pressure. The normal value of PP is approximately 40 mmHg, with an increase above 60 mmHg considered a wide pulse pressure [2]. PP is easy to obtain clinically. PP is a better indicator of functional changes in the arteries than blood pressure [3]. There is a notable correlation between wide pulse pressure and decreased cognitive function [4]. Recent study has also revealed a significant link between PP and the advancement of chronic kidney disease (CKD). PP could potentially serve as a prognostic indicator for the progression of CKD [5]. Several research studies have shown that a wide PP serves as a standalone risk factor for cardiovascular disease [6, 7, 8, 9]. Consequently, effective control of pulse pressure is a critical aspect of blood pressure management. The Knowledge, Attitude, Practice (KAP) model holds significant importance in changing risk factors associated with hypertension, raising awareness and improving behaviour [10] and exploring the effects of KAP in PP. Current hypertension guidelines and antihypertensive treatment targets focus on lowering systolic blood pressure [11], with less attention paid to PP control, which is essential for good health [12]. The objective of this study was to examine the prevalence and associated factors of PP control in hypertensive patients within the context of the KAP model of unified community physician intervention. To explore an appropriate model for integrated management and control of PP in community hypertensive patients, and also to provide new ideas for pulse pressure intervention.

## 2. Basic Materials and Methodology

### 2.1 Research Object

Recruitment was promoted and publicised by community physicians, researchers, community-based medical WeChat groups and posters during the two-month campaign. From 3 January 2020 to 20 January 2020, patients with hypertension were recruited and enrolled in the community of Shenyang City. A total of 3791 patients with hypertension were enrolled in the study, of whom 2660 were eligible and participated in the assessment, which included blood pressure monitoring, physical evaluations, and the completion of questionnaires. All participants were required to provide written informed consent, after having been informed of the purpose, potential benefits, associated medical issues, and the handling of personal information. In the event that a participant was illiterate, written informed consent was obtained from a duly authorised representative. The study was subjected to a review by the Medical Science Research Ethics Committee of the First Affiliated Hospital of China Medical University (reference number [2018] 194).

#### 2.1.1 Inclusion Criteria

(1) Aged 65 years and over;

(2) Hypertension clinical diagnosis;

(3) No communication barriers;

(4) Permanent residents of Shenyang city (residence time ≥6 months);

(5) Voluntarily participate in this project and agree to complete the follow-up, and patients and their families sign an informed consent form.

#### 2.1.2 Exclusion Criteria

(1) An individual with a severe mental illness who is unable to communicate with the investigator;

(2) An individual with a severe physical illness who is unable to cooperate with the investigator.

### 2.2 Research Methods

#### 2.2.1 Experimental Design

2.2.1.1 Training and Management Practices for Physicians

Following the approval of the training materials, the affiliated community physicians underwent a seven-day training programme led by the cardiologists of the First Affiliated Hospital of China Medical University. The training programme consisted of three distinct elements: Diagnostic criteria for hypertension, measurement of blood pressure, and handling of hypertension-related knowledge and behavioural interventions. In particular, the following criteria had to be met: the diagnosis of hypertension was established by systolic blood pressure (SBP) ≥140 mmHg and/or diastolic blood pressure (DBP) ≥90 mmHg, a previous diagnosis of hypertension, or current use of antihypertensive medication. The method for measuring blood pressure entailed the assessment of the systolic and diastolic pressures in the right forearm. This was conducted in a seated position, with an interval of two minutes between each measurement. The mean of the three recorded readings was then calculated to determine the individual’s blood pressure. This process was facilitated by an electronic blood pressure monitor, specifically the Omron (20150061, Omron (Dalian) Co., Ltd., Dalian, China) 1200 U. It was imperative to have a thorough grasp of the various risk factors linked to hypertension, including age, gender, excessive weight, smoking, high salt consumption and lack of physical activity. The dissemination of hypertension-related knowledge to patients in their daily lives was an essential component of the hypertension knowledge dissemination. This was achieved through various methods, including the use of mobile phone notifications, the distribution of pamphlets, and the delivery of popularisation lectures. The objective of these strategies was to facilitate patient comprehension and acceptance of the knowledge being conveyed. Behavioural interventions were used to describe the introduction of strategies designed to address unhealthy lifestyle choices, including the consumption of tobacco, alcohol, and excessive sodium, as well as a lack of physical activity. The involvement of family members in the supervision of patients was encouraged, and patients who demonstrated positive behavioural changes were offered material incentives as a means of maintaining these behaviours over time. The intervention programme for medication was that community physicians guided patients on the use of medication according to the 2018 China Hypertension Prevention and Control Guidelines, complied with the patients’ medication behaviour, and guided the use of antihypertensive medication according to the patients’ wishes. The end of training for community physicians involved having satisfactory results in the included assessment. The management of community physicians was conducted by cardiologists from the First Affiliated Hospital of China Medical University, who visited each community on a regular basis to provide guidance and supervision. The effectiveness of PP control was also employed as a performance evaluation metric and as a criterion for determining incentive grant fees for community physicians.

2.2.1.2 Specific Management of Hypertensive Patients

(1) Individuals who meet the eligibility criteria were enrolled on the KAP model of intervention under the supervision of the unified community physician for a period of one year, from January 2020 to December 2020.

(2) Upon completion of the baseline assessment of the study population, each community physician formulated a pertinent management plan.

(3) The participants in the study underwent a comprehensive evaluation of their blood pressure, individual lifestyle practices, and pertinent factors influencing blood pressure management, as indicated by the initial survey findings.

(4) The KAP Model: Community physicians provided patients with information about hypertension through the use of knowledge bulletin boards, brochures, and lectures. The content encompassed specifics regarding the diagnostic criteria for high blood pressure, advised daily sodium intake, the significance of adhering to prescribed antihypertensive medication, and methods for hypertension prevention. They encouraged patients to reduce their consumption of tobacco and alcohol, to limit their intake of salt, and to engage in more physical activity. The following measures were employed: Patients were scheduled to undergo a sequence of evaluations at specific intervals, which encompassed an examination of the diagnostic criteria for hypertension, an evaluation of daily sodium consumption, and an investigation into the risk factors influencing the management of hypertension. The data obtained from the patients’ responses was subjected to analysis, following which the patients were managed in accordance with the findings. Community physicians used internet-based tools such as WeChat or phone calls for the daily management and monitoring of hypertensive patients. This included tasks such as providing medication reminders, sharing information about hypertension, facilitating physician-patient communication, and answering medication questions. Community physicians improved management efficiency through information technology. Community physicians instructed patients in the methodology of self-monitoring their blood pressure at home, requesting that they performed this task at least twice per week. The results of the PP measurements were conveyed to the community physician via the WeChat group on a weekly basis, with particular attention paid to those who failed to pass the blood pressure monitoring. Patients were advised to regularly monitor their PP or to attend a scheduled assessment and guidance session at the community hospital. In the event that this was necessary, the patients were managed at home. Community healthcare providers distributed complimentary 2 g salt spoons to individuals with hypertension, along with guidance on monitoring salt levels in common condiments for effective salt intake management. For the management of patients who smoked and drank alcohol, it was recommended that poor living habits were improved, the frequency and quantity of tobacco and alcohol consumption were reduced, and the patient’s family was mobilised to perform effective monitoring.

(5) Blood pressure measurements as well as face-to-face questionnaires were carried out by community doctors and relevant researchers at the mid-intervention (month 6) and end-intervention (month 12) periods.

(6) Collection of information: Blood pressure was determined by a calibrated electronic sphygmomanometer (Omron 1200 U). A minimum of five minutes of rest was required prior to the measurement of blood pressure. The blood pressure measurements were conducted under the supervision of a qualified community physician, who oversaw the process of taking blood pressure readings three times on the right upper arm while the individual was seated, with each measurement taken at two-minute intervals. The average of the three readings was recorded as the subject’s systolic and diastolic blood pressure. In order to ensure the integrity of the data collected, it was essential that the investigator conducting the questionnaire investigated employed rigorous quality monitoring procedures. This entailed double-checking any data that might be questionable and subsequently reviewing it with another investigator. Finally, the data must be collated and entered into the appropriate format. The questionnaire comprised a series of questions pertaining to the demographic characteristics of the study participants, including gender, age, community affiliation, height, and weight. Knowing: whether the diagnostic criteria for hypertension and the daily salt intake were identified. Attitude: whether individuals are knowledgeable about the prevention of hypertension. Practice: whether it was due to forgetfulness, poor adherence to medication for side effects, reducing smoking, alcohol consumption, salt intake and increasing physical activity.

#### 2.2.2 Diagnostic Criteria

(1) Hypertension is characterized by an individual having a mean SBP ≥140 mmHg and/or a mean DBP ≥90 mmHg, a documented history of clinically confirmed hypertension, or being on antihypertensive medication within the preceding two weeks. (2) PP control was defined as pulse pressure <60 mmHg. The uncontrolled pulse pressure was defined as pulse pressure ≥60 mmHg. (3) Comorbid diabetes mellitus was characterized as the presence of diabetes mellitus diagnosed before the enrollment of patients with hypertension in the research. (4) Comorbid renal disease was defined as renal disease diagnosed prior to inclusion of hypertensive patients in the study. (5) Body mass index (BMI) <18.5 kg/m^2^ was considered low body mass, BMI ≥18.5 and <24.0 kg/m^2^ was considered normal, BMI ≥24.0 and <28.0 kg/m^2^ was considered overweight, and BMI ≥28.0 kg/m^2^ was considered obese. (6) Normal daily salt consumption was defined as ≤6 g/d. (7) Awareness of hypertension prevention was defined as the patient answering in the affirmative at the time of the questionnaire whether or not hypertension was preventable. (8) Reduction of smoking was defined as giving a positive answer on the questionnaire about whether or not to reduce smoking. (9) Reduced drinking was defined as answering in the affirmative when asked whether or not they had reduced their drinking. (10) Reduction of salt intake was defined as an affirmative response to the questionnaire about whether they had reduced their salt intake. (11) Increased physical activity was defined as a positive response to the question about increased physical activity at the time of the questionnaire.

#### 2.2.3 Statistical Methods

Data were analysed using SPSS 26 software (IBM Corp., Armonk, NY, USA). PP values pre- and post-intervention were presented as mean ± standard deviation ($\bar{x}$ ± s). Group comparisons were conducted utilising the paired samples *t*-test. Knowledge and belief behaviours before and after the intervention were reported as [n (%)], and group comparisons were conducted utilising the χ^2^ test. The factors influencing the effectiveness of PP control in the post-intervention period were subjected to univariate analysis, followed by multivariate analysis.

## 3. Results

### 3.1 Baseline Data

A total of 3791 individuals were enrolled in this study in January 2020. Of these, 2660 were aged ≥65 years, with a 100% follow-up rate. The age range of this cohort was 65–85 years, with a mean age of (71.17 ± 5.11) years. The population was comprised of 37.07% males and 62.93% females. The age subgroups of patients aged 65–79 years and those aged 80 years and above constituted 90.64% and 9.36%, respectively, of the total population in their respective age groups. The BMI subgroups of low body mass, normal, overweight and obese accounted for 0.90%, 33.08%, 47.22% and 18.80%, respectively. A total of 22.56% of the population exhibited comorbid diabetes, while 77.44% demonstrated non-comorbid diabetes. A prevalence of 0.53% was observed for comorbid kidney disease, while 99.47% of individuals exhibited no comorbid kidney disease. The study population consisted of 67.18% people who had never smokered and 32.82% smokers. Meanwhile, there were 61.39% of people who had never drunk alcohol and 38.61% of alcohol drinkers, as outlined in Table 1.

**Table 1.** **Baseline data (n = 2660)**.

Items	Divide into groups	Number (n)	Component ratio (%)
Genders	male	986	37.07
	female	1674	62.93
Age	65∼79	2411	90.64
	≥80	249	9.36
BMI	low body weight	24	0.90
	normality	880	33.08
	overweight	1256	47.22
	obesity	500	18.80
Whether there is a combination of diabetes	yes	600	22.56
	no	2060	77.44
Whether there is a combination of kidney diseases	yes	14	0.53
	no	2646	99.47
Whether you smoke or not	yes	873	32.82
	no	1787	67.18
Whether you drink alcohol or not	yes	1027	38.61
	no	1633	61.39

BMI, body mass index.

### 3.2 Pre- and Post-intervention KAP Pulse Pressure Change

The mean pulse pressure of the patients prior to the intervention was 67.42 ± 15.47 mmHg, while the mean pulse pressure of the patients following the intervention was 56.71 ± 11.60 mmHg. The mean pulse pressure decreased by 10.71 mmHg (95% CI: 10.09, 11.33 mmHg), and the difference was statistically significant (t = 33.79, *p* < 0.01). Furthermore, the hypertensive population was stratified according to age, with categories of 65~79 years and ≥80 years, and PP changes were evaluated pre- and post- intervention in the different age groups. After the intervention, PP decreased by 10.48 mmHg, 95% CI (9.83, 11.13) mmHg in those aged 65~79 years compared to pre-intervention, which was statistically significant (t = 31.61, *p* < 0.01). After the intervention, PP decreased by 13.01 mmHg, 95% CI (10.88, 15.12) mmHg in those ≥80 years of age compared with the pre-intervention, the difference was statistically significant (t = 12.09, *p* < 0.01), as outlined in Table 2.

**Table 2.** **Changes in PP pre- and post-intervention**.

Items	Pre-intervention	Post-intervention	Difference	t-value	*p*-value
	($\bar{x}$ ± s, mmHg)	($\bar{x}$ ± s, mmHg)	(95% CI, mmHg)		
General	67.42 ± 15.47	56.71 ± 11.60	10.71, (10.09, 11.33)	33.79	<0.01
65∼79	67.06 ± 15.44	56.58 ± 11.52	10.48, (9.83, 11.13)	31.61	<0.01
≥80	70.94 ± 15.28	57.94 ± 12.30	13.01, (10.88, 15.12)	12.09	<0.01

PP, pulse pressure.

### 3.3 Changes in KAP Pre- and Post-intervention

A comparison of the KAP of the unified community physician intervention was conducted before and after the intervention. The results demonstrated a significant increase in awareness of hypertension-related knowledge and prevention, medication and behavioural adherence following the implementation of the intervention, when compared with the pre-intervention period. The difference was statistically significant, as outlined in Table 3.

**Table 3.** **Changes in KAP pre- and post-intervention**.

Items			Pre-intervention	Post-intervention	χ^2^-value	*p*-value
			N, (%)	N, (%)		
Knowledge and awareness of hypertension prevention						
	Whether the criteria for the diagnosis of hypertension are known	yes	1354 (50.90)	2334 (87.74)	848.89	<0.01
		no	1306 (49.10)	326 (12.26)		
	Whether or not the daily amount of salt is known	yes	1399 (52.59)	2526 (94.96)	1234.09	<0.01
		no	1261 (47.41)	134 (5.04)		
	Whether they are aware of hypertension prevention	yes	1422 (53.46)	2514 (94.51)	1164.57	<0.01
		no	1238 (46.54)	146 (5.49)		
Medication and behavioural adherence						
	Whether it is due to poor adherence to forgotten medication	yes	1152 (43.31)	368 (13.83)	566.13	<0.01
		no	1508 (56.69)	2292 (86.17)		
	Whether it is due to poor adherence to medication for adverse effects	yes	637 (23.95)	83 (3.12)	492.99	<0.01
		no	2023 (76.05)	2577 (96.88)		
	Whether or not to reduce smoking	yes	253 (28.98)	676 (77.43)	411.61	<0.01
		no	620 (71.02)	197 (22.57)		
	Whether or not to reduce alcohol consumption	yes	196 (19.08)	625 (60.86)	373.43	<0.01
		no	831 (80.92)	402 (39.14)		
	Whether or not to reduce salt intake	yes	731 (27.48)	2361 (88.76)	2051.79	<0.01
		no	1929 (72.52)	299 (11.24)		
	Whether or not to increase physical activity	yes	804 (30.23)	2377 (89.36)	1934.62	<0.01
		no	1856 (69.77)	283 (10.64)		

KAP, Knowledge, Attitude, and Practice.

### 3.4 Analysis of Factors Influencing the Effectiveness of PP Control at the Post-intervention

Following the implementation of the KAP model under the unified society physician intervention for a period of one year, the pulse pressure control rate increased from 32.59% (867/2660) to 64.92% (1727/2660) in the cohort of hypertensive patients recruited at baseline. Factors affecting PP control were studied, with factors including baseline information, knowledge about hypertension, awareness of prevention and medication and behavioural adherence. The results showed that there were differences between patients in the PP control group and the uncontrolled group at the end of the intervention in terms of age, taking antihypertensive medication, whether they mastered the diagnostic criteria of hypertension, whether they mastered the daily amount of salt, whether they had an awareness of hypertension prevention, whether they had poor adherence to medication due to adverse reactions, whether they reduced smoking, whether they reduced drinking, whether they reduced the daily amount of salt intake, and whether they increased physical activity, but the differences were not statistically significant (*p* > 0.05). There were differences in gender, history of comorbid diabetes, history of nephropathy, and whether they had poor adherence to medication due to forgetfulness, and the differences were statistically significant (*p* < 0.05), as outlined in Table 4.

**Table 4.** **A single-factor analysis of the effectiveness of PP control at the end of the intervention**.

Items			Post-intervention		χ^2^-*value*	*p*-value
			yes	no		
Basic data						
	genders	female	1050 (62.72)	624 (37.28)	9.61	<0.01
		male	677 (68.66)	309 (31.34)		
	age	65∼79	1577 (65.41)	834 (34.59)	2.65	0.10
		≥80	150 (60.24)	99 (39.76)		
	history of comorbid diabetes	yes	362 (60.33)	238 (39.67)	7.17	<0.01
		no	1365 (66.26)	695 (33.74)		
	history of nephropathy	yes	5 (35.71)	9 (64.29)	4.06	0.04
		no	1722 (65.08)	924 (34.92)		
	taking antihypertensive medication	not taking medication	634 (65.56)	333 (34.44)	2.72	0.26
		taking 1 medication	990 (65.17)	529 (34.83)		
		taking ≥2medication	103 (59.20)	71 (40.80)		
Knowledge and awareness of hypertension prevention						
	whether they mastered the diagnostic criteria of hypertension	yes	1508 (64.61)	826 (35.39)	0.83	0.36
		no	219 (67.18)	107 (32.82)		
	whether they mastered the daily amount of salt	yes	1639 (65.67)	887 (35.11)	0.04	0.85
		no	88 (65.67)	46 (34.33)		
	whether they had awareness of hypertension prevention	yes	1630 (64.84)	884 (35.16)	0.16	0.69
		no	97 (66.44)	49 (35.56)		
Medication and behavioural adherence						
	whether they had poor adherence to medication due to forgetfulness	yes	209 (56.79)	159 (43.21)	12.40	<0.01
		no	1518 (66.23)	774 (33.77)		
	whether they had poor adherence to medication due to adverse reactions	yes	46 (55.42)	37 (44.58)	3.40	0.07
		no	1681 (65.23)	896 (34.77)		
	whether they reduced smoking	yes	440 (65.09)	236 (34.91)	0.09	0.96
		no	126 (63.96)	71 (36.04)		
		never smoking	1161 (64.97)	626 (35.03)		
	whether they reduced drinking	yes	408 (65.28)	217 (34.72)	2.15	0.34
		no	273 (67.91)	129 (32.09)		
		never drinking	1046 (64.05)	587 (35.95)		
	whether they reduced the daily amount of salt intake	yes	1534 (64.97)	827 (35.03)	0.02	0.86
		no	193 (64.55)	106 (35.45)		
	whether they increased physical activity	yes	1547 (65.08)	830 (34.92)	0.24	0.62
		no	180 (63.60)	103 (36.40)		

#### Multi-factor Analysis of the Control Effect of End-stage Pulse Pressure

The control of PP was taken as the dependent variable (controlled pulse pressure: Y = 0, uncontrolled pulse pressure: Y = 1), and the gender with statistical significance in the univariate analysis, whether there was a history of diabetes mellitus, whether there was a history of kidney disease, and whether there was poor compliance due to forgetting medications were taken as independent variables. Multiple logistic regression analysis was employed. The study findings indicated that being female, having a history of diabetes, and demonstrating poor compliance with medication adherence were identified as risk factors for controlling pulse pressure (all *p* < 0.05), as outlined in Table 5.

**Table 5.** **Multi-factor analysis of the control effect of PP at the end of intervention**.

Items		β-value	Waldχ^2^-value	*p*-value	OR value	OR-value (95% CI)
Genders	male				1.00	
	female	0.264	9.53	<0.01	1.30	(1.10, 1.54)
History of comorbid diabetes	no				1.00	
	yes	0.260	7.24	<0.01	1.30	(1.07, 1.57)
History of comorbid nephropathy	no				1.00	
	yes	1.091	3.75	>0.05	2.98	(0.99, 8.99)
Whether they had poor adherence to medication due to forgetfulness	no				1.00	
	yes	0.431	12.96	<0.01	1.51	(1.21, 1.89)

OR, odds ratio.

## 4. Discussion

With age leading to vascular stiffness, more than two-thirds of people over the age of 65 suffer from hypertension [13]. Hypertension is a heavy burden on global health, and every country is actively exploring cost-effective hypertension prevention and management models. A large number of studies at home (South Asia and China) and abroad have confirmed the effectiveness of community-based hypertension management [14, 15, 16]. As a result, more and more countries are moving hypertension prevention and management into the community. Reduced drinking was defined as answering in the affirmative when asked whether or not they hadreduced their drinking [17, 18], but the management of pulse pressure was weak. The 2018 European guidelines on blood pressure have extensively discussed PP as a risk marker, confirming that PP ≥60 mmHg in older adults with hypertension increases cardiovascular risk [2]. Therefore, the management of primary prevention in elderly individuals with hypertension should be given due consideration.

Franklin SS *et al*. [19] studied a 23% increased risk of coronary heart disease for every 10 mmHg increase in PP. Haider AW *et al*. [20] increased the risk of heart failure by 55% for every 16 mmHg increase. Each 10 mmHg increase in PP increased the risk of stroke by 11% and all-cause mortality by 16% [21]. In addition, the adjusted hazard ratio for the development of atrial fibrillation was 1.28 for each 20 mmHg increase in PP [22]. Our study showed that under unified community physician management, the pulse pressure difference decreased by 10.71 mmHg, 95% CI (10.09, 11.33) mmHg, and the PP control rate increased from 32.59% at baseline to 64.92%. In addition, we found differences in PP levels before and after intervention in patients with hypertension of different ages (65–79 years, ≥80 years), it was concluded that the KAP management mode under the guidance of community physicians played an important role in the pulse pressure management of elderly hypertensive patients.

Risk factors for hypertension included modifiable factors such as unhealthy diet, lack of exercise, smoking, alcohol abuse, obesity, diabetes and kidney disease. Another was the immutable risk factors, such as family history and age [23]. Understanding and consciousness were significant factors in the management and prevention of hypertension [24]. Reduced drinking was defined as answering in the affirmative when asked whether or not they had reduced their drinking. The study showed that the KAP intervention model led by community physicians can effectively change the risk factors of patients with hypertension. Chuang SY *et al*. [25] shows increased physical activity good for pulse pressure control in the elderly. In patients with hypertension, it was common to not adhere to medication, and the medication adherence of patients with hypertension in this study has improved, but it still needed to be strengthened due to the influence of multiple factors. Therefore, it was necessary to encourage patients to use auxiliary tools, such as a weekly tablet dispenser and medication alarm device [26].

The post-intervention control of pulse pressure was initially subjected to univariate analysis, followed by multivariate analysis of significant variables. The findings revealed that being female, having a history of diabetes, and poor medication adherence due to forgetfulness were identified as risk factors for pulse pressure control (*p* < 0.05). The risk factor of pulse pressure control differs between genders, a finding consistent with the study by Jia *et al*. [27]. It is hypothesized that postmenopausal women may lose the cardiovascular protective effects of estrogen. However, as we did not statistically analyze the menopausal status of females in our sample, definitive conclusions cannot be drawn, warranting further investigation. Research indicates that both hypertension and diabetes can lead to a decline in memory function. Hypertension reduces the functional connectivity of the hippocampus and damages the integrity of brain white matter, thereby impairing the memory function of hypertensive patients [28, 29]. Werhane *et al*. [30] further demonstrated that reducing pulse pressure can prevent and delay brain aging. Type 2 diabetes can cause a rapid decline in memory function, with study suggesting vascular factors as the main cause, while recent research suggests that insulin resistance may also play a direct role. Poor blood pressure control in patients with hypertension and diabetes leads to difficulties in controlling pulse pressure [31]. The pharmacological management of hypertension serves as the cornerstone therapy in the treatment of high blood pressure, as inadequate adherence to antihypertensive medications poses a significant challenge in controlling hypertension. In light of this, five key strategies are considered to enhance medication adherence: (1) The increased utilization of online-based telemedicine platforms, such as WeChat and SMS, for reminding patients to take medication (e.g., reminders 2–3 times a day) has been noted in recent study [32]. (2) Increasing the frequency of follow-up visits or consultations can lead to heightened awareness of the disease among patients and improve medication adherence [33]. (3) Improving physician prescriptions: Research indicates that thiazide diuretics can more effectively achieve pulse pressure control [12]. Additionally, it is advisable to recommend patients switch to combination antihypertensive drugs to reduce the actual quantity of medications taken, thereby enhancing medication adherence. (4) Collaborating with the family members of hypertensive patients, administering medication under their supervision, providing patients with emotional and lifestyle support, and simultaneously offering community physicians more comprehensive and precise information, represent vital therapeutic measures [9]. (5) For patients for whom it is challenging for family members to provide daily reminders to take medication, we propose the acquisition or distribution of compartmentalized pill boxes by community physicians. Each box is designated for a specific mealtime, enhancing medication adherence and subsequently improving blood pressure control among patients. Therefore, it is imperative to actively promote awareness of knowledge pertaining to hypertension and integrate such behaviors into daily life to cultivate long-term habits.

## 5. Conclusions

In conclusion, a unified intervention by community physicians can change the perceptions of elderly hypertensive patients, improve medication adherence and modify poor lifestyle habits, thereby improving pulse pressure control in elderly hypertensive patients in the community. It should be noted that the study was subject to the following limitations: (1) It does not assess the economic benefits of research. (2) It does not document events related to coronary heart disease. (3) The study suffers from recall bias.

## Availability of Data and Materials

The data did not reproduce material from other sources. The raw data of the present study will be made available after evaluation and permission by the subject principals. Requests to access the data should be directed to the corresponding author.
